# The mechanism of parvalbumin interneurons regulating glutamatergic neurons involvement in stress induced anxiety in the basolateral amygdala of male mice

**DOI:** 10.1038/s41598-025-10130-x

**Published:** 2025-07-21

**Authors:** Xiaorui Su, Xuetong Dong, Chao-Long Lu, Minglong Zhang, Yaping Li, Han Xiao, Jian Wang, Yufei Sun, Bin Cong, Songjun Wang

**Affiliations:** 1 Hebei Key Laboratory of Forensic Medicine, Collaborative Innovation Center of Forensic Medical Molecular Identification, Research Unit of Digestive Tract Microecosystem Pharmacology and Toxicology, Chinese Academy of Medical Sciences, College of Forensic Medicine, Hebei Medical University, No. 361 Zhong Shan Road, Shijiazhuang, Hebei China; 2 Department of Genetics, College of Basic Medicine, Qiqihar Medical University, No. 333 Bukui South Street, Qiqihar, 161006 Heilongjiang China; 3 Hainan Tropical Forensic Medicine Academician Workstation, Haikou, 571199 Hainan Province China

**Keywords:** Acute stress, Chronic stress, Stress induced anxiety, PV-INs, Glutamatergic neurons, Cell biology, Neuroscience

## Abstract

Modern life’s fast-paced and the unexpected conditions contribute to escalating stress levels, often leading to anxiety disorders and posing significant challenges to physical and mental health. In judicial practice, the parties often suffer from anxiety disorder under the great stress. However, the precise mechanisms underlying stress-induced anxiety disorders remain incompletely understood. This study aims to explore the neural mechanisms by which stress-induced imbalances in the basolateral amygdala (BLA) parvalbumin interneurons (PV-INs) and glutamatergic neurons lead to anxiety. This study used behavioral analysis, morphology, patch clamp electrophysiology, and viral interference techniques to detect the number of BLA PV-INs and glutamatergic neurons, as well as the excitability of glutamatergic neurons. Results demonstrated that acute and chronic stress adversely affect PV-INs in the BLA, diminishing their numbers and resulting in glutamatergic neurons disinhibition, thereby enhancing glutamatergic neurons excitability and precipitating anxiety behaviour. The anxiety disorder can be effectively improved by activating PV-INs. This study reveals the mechanism of internal amygdala PV-INs regulation leading to anxiety disorders under acute and chronic stress.

## Introduction

Anxiety disorder stands as the most prevalent mental illness, affecting approximately 14% of the global population, with a higher incidence among women^1–4^. However, in judicial practice, it has been found that in the face of interrogation, assault, traumatic life events and other situations, the proportion of male anxiety disorders is higher, which is also a group that cannot be ignored. Chronic stress and acute traumatic events are recognised as independent risk factors for anxiety disorders. However, due to an incomplete understanding of the mechanisms by which stress induces anxiety, precise treatment options remain limited^5–7^, meanwhile, it is also difficult to identify in judicial practice. Therefore, elucidating the stress-induced anxiety mechanism is imperative for developing targeted interventions and accurate identification.

Anxiety behaviour is intricately modulated by a neural network spanning various brain regions, including the basolateral amygdala (BLA), hippocampus and prefrontal cortex^3,8,9^. Among these, the BLA is considered a pivotal region in stress response mediation^10–13^. Comprising primarily excitatory pyramidal neuron and approximately 20% GABAergic interneurons, the BLA’s proper function hinges on the delicate balance between inhibitory and excitatory signals. Hyperactivation of BLA glutamatergic neurons due to disinhibition is widely considered the neural basis of stress-induced anxiety^14,15^. GABAergic interneurons interact with excitatory glutamatergic neurons through feedforward inhibition, feedback inhibition and other forms to form intricate neural microcircuits regulating BLA function and anxiety behaviour^16,17^. Previous studies have shown^18^ that chronic stress can lead to degeneration and death of GABAergic interneurons in the BLA and decrease in number, triggering an imbalance in excitatory and inhibitory signalling and resultant anxiety-like behaviours. However, the specific role of GABAergic interneurons in stress-induced anxiety disorders within the BLA remains incompletely understood.

Parvalbumin interneurons (PV-INs) constitute approximately 40% to 50% of GABAergic interneurons and are particularly susceptible to stress-induced injury^19^. PV-INs intricately innervate the cell body, axon initiation and apical dendrites of glutamatergic neurons, exerting a predominate inhibitory influence around glutamatergic neurons bodies^20,21^. PV-INs can regulate excitatory/inhibitory balance, and play an important role in anxiety, post-traumatic stress disorder, schizophrenia and autism. PV neurons in the BLA play crucial roles in the expression of fear and anxiety. Understanding how these neurons regulate these stress related behaviors is important. Given the importance of these neurons in anxiety, not surprisingly, a lot of studies have examined their roles using various stress models. Indeed, decreased number of PV cells have been associated with anxiety (Urakawa et al. 2013). Enhancing and inhibiting PV neuronal activity by DREADD have been shown to decrease and increase anxiety in mice, respectively (Luo et al. 2019; Asim et al. 2024). Fu et al. (2022) also showed that PV could modulate the activity of excitatory neurons and brain oscillation in the BLA. Although the effect of stress and PV neuronal modulation on excitatory neuronal activity seems novel, the current study lacks very novel findings to reveal additional effect of PV neurons in modulating anxiety that has not been shown in these previous studies^22^. Nevertheless, the precise mechanism by which PV-INs in the BLA regulate excitatory pyramidal neuron in stress-induced anxiety remains elusive.

This study employs behavioural, morphological and electrophysiological assessments to reveal that both acute and chronic stress diminish PV-INs in the BLA, heighten glutaminergic neurons excitability, and induce anxiety-like behavioural changes. Moreover, utilising chemogenetic methods to modulate PV-INs, we investigate subsequent changes in glutaminergic neurons excitability and anxiety-like behaviour. This study aims to uncover the role of PV-INs in anxiety disorders stemming from acute and chronic stress, providing the basis for judicial practice identification and insights for clinical interventions.

## Results

### Acute and chronic stress led to anxiety-like behaviours in mice

**Fig. 1:**
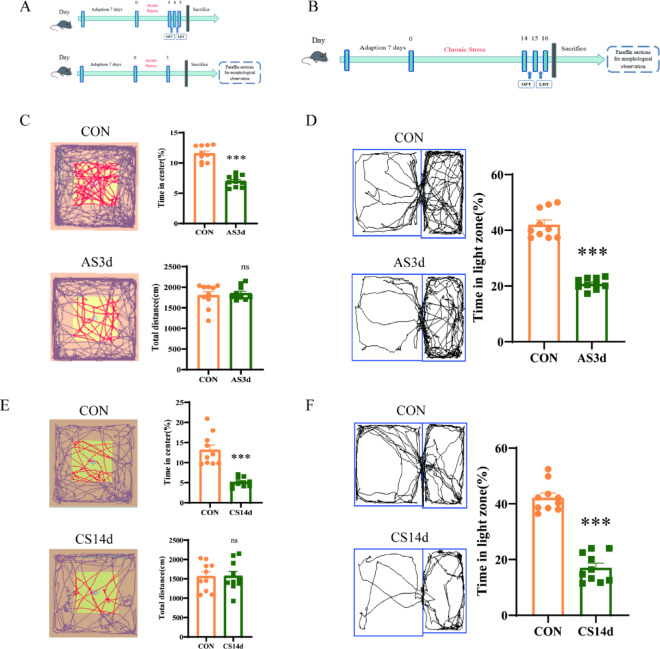
Anxiety-like behaviours in mice induced by acute and chronic stress. (**A**) The experimental process of behavioural monitoring of acute stress animal models. After behavioural monitoring, the animals were euthanised. Subsequently, paraffin sections were prepared for morphological observations. (**B**) Behavioural monitoring of chronic stress animal models. (**C**) The central region activity time ratio decreased in the acute stress group (n = 10, ***P < 0.001 vs. CON group), while the total moving distance showed no significant difference compared to the control group (*P* = 0.7766 vs. CON group). (**D**) The percentage of active time of the light box in the acute stress group was reduced (n = 10, ***P < 0.0001 vs. CON group). (**E**) The central region activity time ratio decreased in the chronic stress group (n = 10, ***P < 0.001 vs. CON group), with no significant difference in the total moving distance compared to the control (*P* = 0.8258 vs. CON group). (**F**) Decreased percentage of active time of the light box in chronic stress group (n = 10, ***P < 0.0001 vs. CON group). The data are presented as mean ± SEM.

Behavioural changes in mice induced by acute and chronic stress were evaluated using the light–dark box shuttle test and open field test (Fig. 1A,B). In the acute stress group, the open field test and the light–dark box shuttle test began on the 4th and 5th day respectively. In the acute stress group (AS3d), mice displayed reduced exploration behaviour in the central region compared to the control group (CON) (Fig. 1C, Unpaired *t test, t* = 5.3790*, P* < 0.001). Conversely, there was no significant difference in total activity distance in the AS3D group compared to the CON group (Unpaired *t test, t* = 0.2915*, P* = 0.7766). In the light and dark box shuttle experiment, the percentage of light box activity time of mice in the acute stress group was significantly reduced compared to the CON group (Fig. 1D, Unpaired *t test, t* = 11.40*, P* < 0.0001). Collectively, these findings indicate that acute stress can reduce the exploration behaviour of the central region and light box in mice. The results are basically consistent with previous studies on zebrafish^7^.

Similarly, in the chronic stress group (CS14d), the open field test was conducted on the 15th day, whereas the light–dark box shuttle test was conducted on the 16th day. In the open field test, the central activity time of CS14d mice was lower than that of the CON group (Fig. 1E, Unpaired *t test, t* = *4.623, P* < 0.001), with no significant difference in total activity distance between the chronic stress group and CON group (Unpaired *t test, t* = 0.2260*, P* = 0.8258). In the light and dark box shuttle experiment, the activity time of mice in the chronic stress group was significantly reduced compared to the CON group (Fig. 1F, Unpaired *t test, t* = 8.1670*, P* < 0.0001). These results indicate that chronic stress can lead to a decrease in the exploration behaviour of the central region and light box in mice. The results are basically consistent with previous studies^7,18,23^**.** Overall, these behavioural experiments reveal that both acute and chronic stress can induce anxiety-like behaviour in mice.

### Acute and chronic stress led to a decrease in PV-INs expression whereas an increase in glutamatergic neurons in BLA

**Fig. 2:**
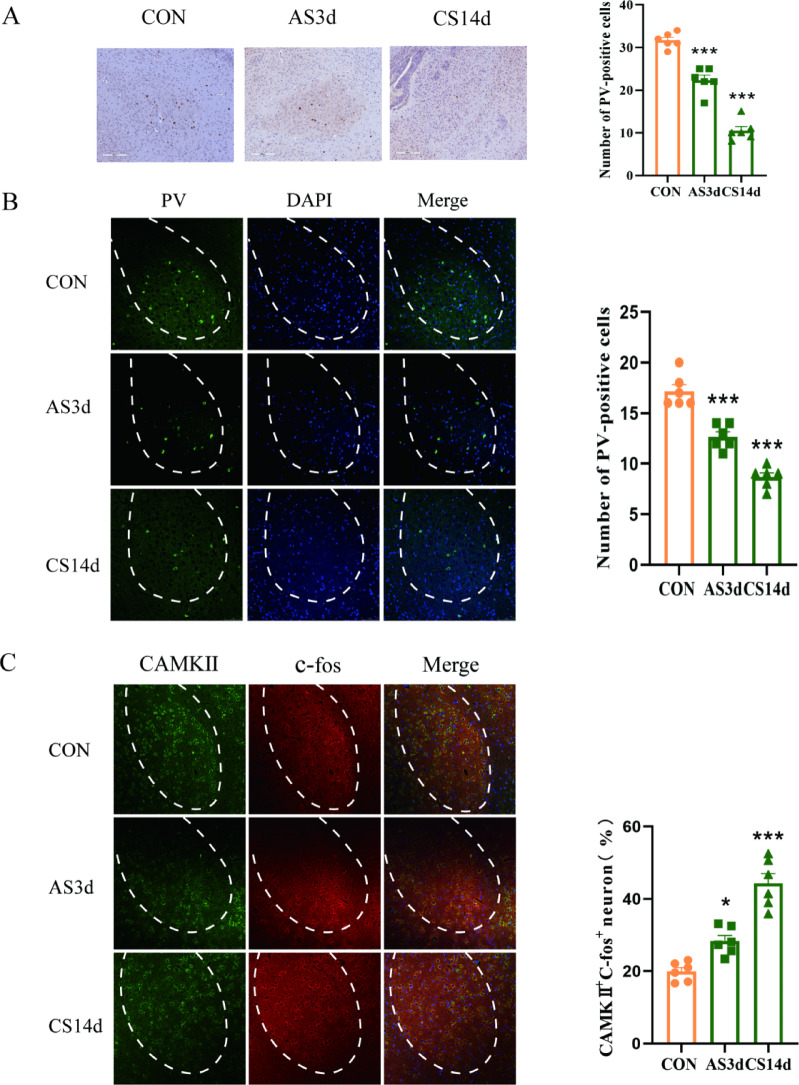
Effect of acute and chronic stress on PV-INs positive expression and glutamatergic neurons. (**A**) The number of positive PV-INs expressions under acute and chronic stress conditions in the BLA was lower than the control at 200 × magnification (n = 6, ***P < 0.0001 vs. CON group). (**B**) The number of positive PV-INs expression in acute and chronic stress in the BLA was lower than that in the control group at 200 × under a fluorescence microscope (n = 6, ***P < 0.0001 vs. CON group). (**C**) The number of CAMKII and c-fos co-expressing cells in the acute and chronic stress groups of the BLA increased compared with the control group at 200 × under a fluorescence microscope (n = 6, *P < 0.05, ***P < 0.0001 vs. CON group). Data are presented as mean ± SEM.

Immunohistochemistry and immunofluorescence techniques were employed to investigate the impact of acute and chronic stress on PV-IN neurons in the BLA. Immunohistochemical analysis revealed a lower number of positive PV-INs in the acute and chronic stress groups compared to the control group (Fig. 2A, one-way ANOVA F_(2, 15)_ = 114.9, *p* < 0.0001). Immunofluorescence results showed a reduction in the number of positive PV-INs expression in both stress groups compared to controls, suggesting a decrease in PV-INs expression following stress (Fig. 2B, one-way ANOVA F_(2, 15)_ = 63.82, *p* < 0.0001).

Previous studies have demonstrated that PV-IN neurons play a role in glutamatergic neurons and affect the excitability of glutamatergic neurons, which triggers various biological functions. CAMK II is a marker protein for glutamatergic neurons. To explore the impact of glutamatergic neurons activation on BLA when the expression of PV-INs neurons is reduced due to acute and chronic stress, immunofluorescence analysed the co-expression of CAMKII and c-fos in the BLA. The results revealed that the number of activated glutamatergic neurons increased in both the acute and chronic stress groups compared to the control group (Fig. 2C, one-way ANOVA F_(2, 9)_ = 37.90,*p* < 0.0001). These results illustrate that acute and chronic stress leads to a decrease in PV-INs expression in the BLA, accompanied by increased glutamatergic neurons activation, suggesting a potential mechanism underlying stress-induced anxiety-like behaviour.

### Acute and chronic stress enhanced excitability of glutamatergic neurons in BLA

**Fig. 3:**
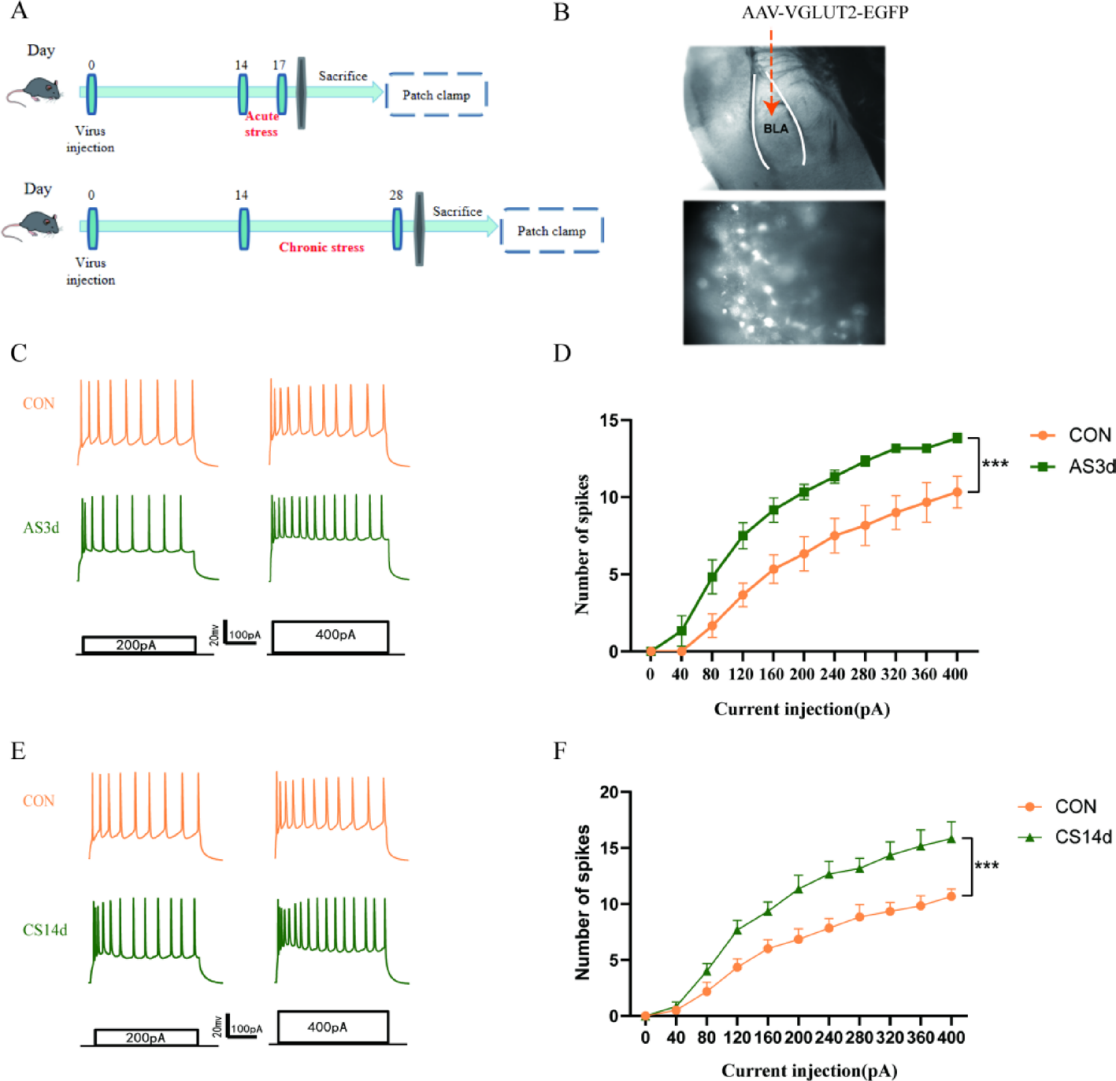
Increased excitability of glutamatergic neurons in the BLA caused by acute and chronic stress. (**A**) The mice were injected with an adeno-associated virus at the end of the adaptation period, and the acute and chronic stress model was established 14 days later. After the modelling was completed, the brain was extracted, and the patch-clamp experiment was performed. (**B**) The BLA structure, adeno-associated virus structure and fluorescence-labelled glutamatergic neurons under 100 × magnification. (**C**) Typical diagram of action potentials induced by glutamatergic neurons 200pA and 400pA current in the BLA region in the acute stress and control groups. (**D**) Significant difference in the discharge rate of glutamatergic neurons injection current-dependent action potential in the BLA region after acute stress. (n = 6 cells from 6 mice, ***P < 0.0001 vs. CON group). (**E**) Typical diagram of action potentials induced by glutamatergic neuron 200pA and 400pA current in the BLA region in the chronic stress and control groups. (**F**) Significant difference in discharge rate of glutamatergic neurons injection current-dependent action potential in the BLA region after chronic stress (n = 6 cells from 6 mice, ***P < 0.0001 vs. CON group). Data are presented as mean ± SEM.

Whole cell patch-clamp technique was employed to explore glutamatergic neurons excitability changes before and after acute and chronic stress. As illustrated in Fig. 3, the mice in the AS3d group and the CS14d group were used in patch clamp experiment after the modelling was completed (Fig. 3A). AAV-VGLUT2-EGFP was injected into the BLA by stereotaxic injection, and fluorescence labelled glutamatergic neurons was identified under the microscope (Fig. 3B). whole cell patch-clamp analysis revealed that the number of APs induced by glutamatergic neurons increased in the acute stress group at a current exceeding 120 pA (Fig. 3C,D, Two-way-ANOVA, F_(1, 110)_ = 87.24, *P* < 0.0001), while the same was observed in the chronic stress group at a current exceeding 200 pA (Fig. 3E,F, Two-way-ANOVA, F_(1,110)_ = 78.24, *P* < 0.0001). These results indicate that acute and chronic stress can lead to an increase in glutamatergic neurons excitability in the BLA.

### Chemogenetic activation of PV-INs reversed stress-induced hyperexcitability in glutamatergic neurons

**Fig. 4:**
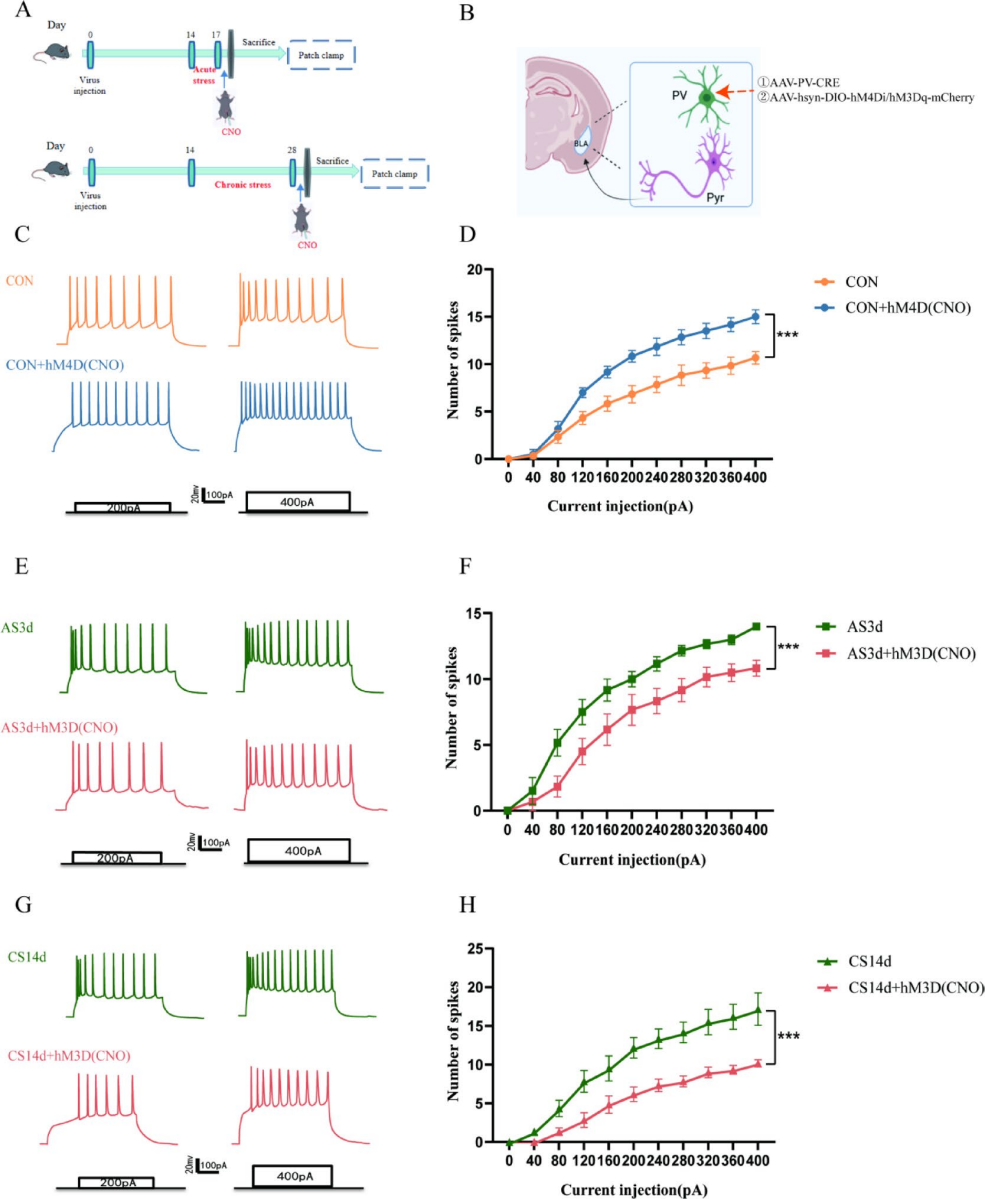
Changes in glutamatergic neurons excitability of PV-INs neurons regulated by chemogenetic regulation. (**A**) After the adaptation period, the mice were injected with an adeno-associated virus, and the acute and chronic stress model was established 14 days later. The mice were activated by an intraperitoneal injection of CNO, 15 min before euthanasia, and a patch-clamp experiment was performed subsequently. (**B**) Structural formula and target of adeno-associated virus. (**C**) Typical diagram of an action potential induced by glutamatergic neurons under 200pA and 400pA stimulation after PV-INs inhibition under physiological conditions. (**D**) The rate of injection current-dependent action potential in glutamatergic neurons significantly increased in CON + hM4D group (n = 6 cells from 6 mice, ***P < 0.0001 vs. CON group). (**E**) Typical diagram of glutamatergic neurons induced action potential under 200pA and 400pA stimulation after PV-INs activation under acute stress conditions. (**F**) The rate of injection current-dependent action potential in glutamatergic neurons significantly increased in AS3d + hM3D group (n = 6 cells from 6 mice, ***P < 0.0001 vs. CON group). (**G**) Typical diagram of action potential induced by glutamatergic neuron under 200pA and 400pA stimulation after PV-INs activation under chronic stress conditions. (**H**) The rate of injection current-dependent action potential in glutamatergic neurons significantly increased in CS14d + hM3D group (n = 6 cells from 6 mice, ***P < 0.0001 vs. CON group), the data are presented as mean ± SEM.

It was determined that the mice developed anxiety-like behaviour after the decrease in PV-INs number and an increase in glutamatergic neurons excitability caused by acute and chronic stress. Moreover, chemogenetic regulation of PV-INs under physiological state, acute stress and chronic stress confirmed that the excitability of glutamatergic neurons in the BLA is associated with a decrease in PV-IN number after acute and chronic stress. The whole cell patch-clamp technique was used to observe the excitability of glutamatergic neurons (Fig. 4A). Furthermore, the structural formula and targets of the adeno-associated virus are presented in Fig. 4B. Electrophysiological analysis revealed an increase in the number of APs induced by glutamatergic neurons under a current stimulation exceeding 160 pA following the inhibition of PV-INs under physiological conditions (Fig. 4C,D, Two-way-ANOVA, F_(1,110)_ = 89.72, *P* < 0.0001). Moreover, the number of APs induced by glutamatergic neurons decreased under a current stimulation exceeding 80 pA following activation of PV-INs under acute stress (Fig. 4E,F, Two-way-ANOVA, F_(1,110)_ = 72.50, *P* < 0.0001). Conversely, under chronic stress conditions, the number of APs induced by glutamatergic neurons under 120 pA current stimulation decreased after activating PV-INs (Fig. 4G,H, Two-way-ANOVA, F_(1,110)_ = 106.5, *P* < 0.0001). These findings reveal that glutamatergic neurons excitability increased after PV-INs inhibition in the physiological state but decreased after PV-INs activation under acute and chronic stress conditions.

### Chemogenetic activation of PV-INs alleviated stress-induced anxiety-like behaviours in mice

**Fig. 5:**
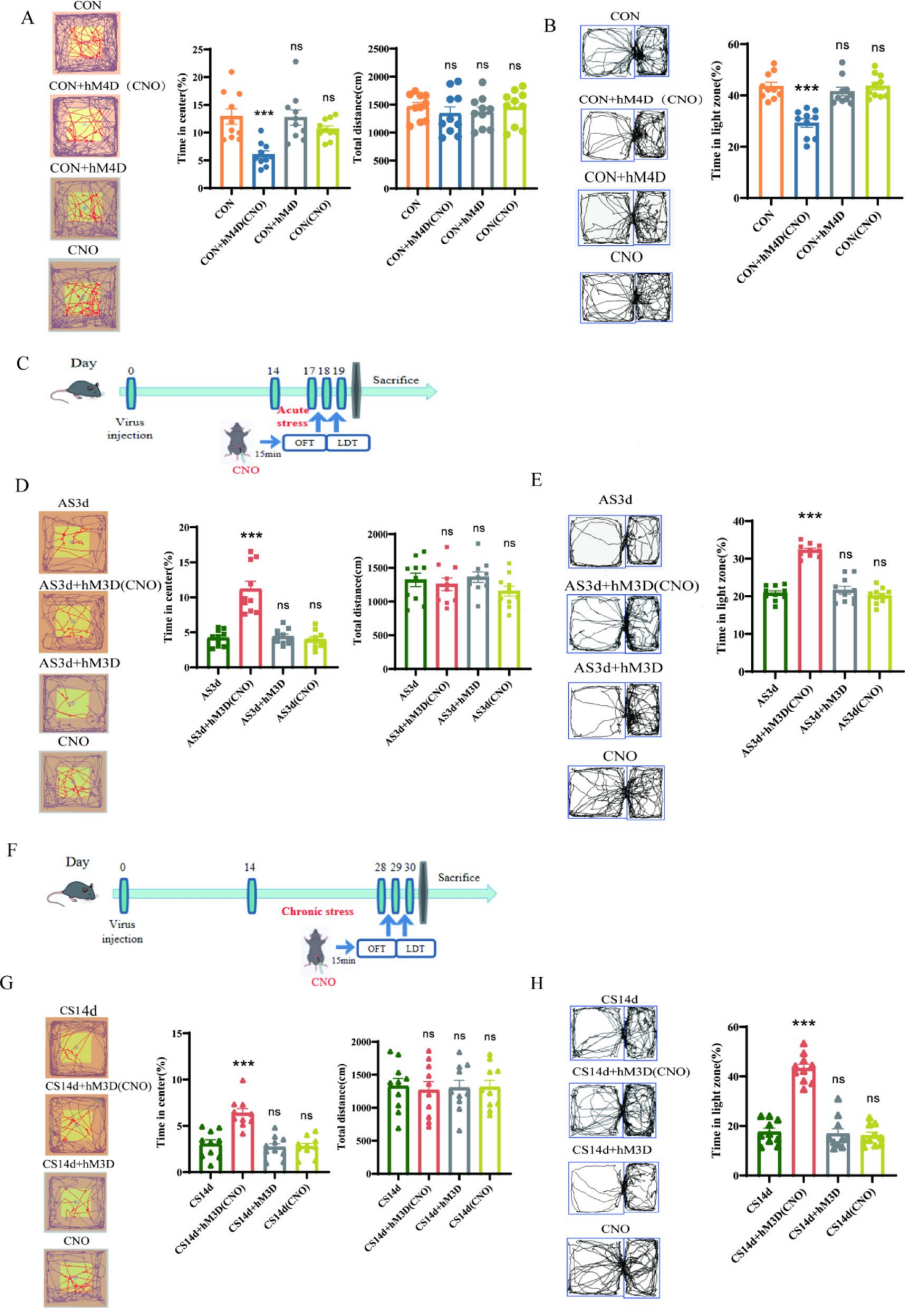
Effect of chemogenetic regulation of PV-INs on mouse behaviour. (**A**) Time in center decreased after the inhibition of PV-INs under physiological conditions (n = 10 from 10 mice per group, **P < 0.01 vs. CON and CON + hM4D groups), with no difference in total distance between the two groups (*P* > 0.05 vs. CON and CON + hM4D groups). (**B**) Time in light zone decreased after the inhibition of PV-INs in the physiological state (n = 10 from 10 mice per group, ***P < 0.001 vs. CON and CON + hM4D groups). (**C**) Acute stress model was established 14 days after virus injection, and behavioural monitoring of CNO activation was performed 15 min before intraperitoneal injection. (**D**) Time in center increased after the activation of PV-INs under acute stress conditions (n = 10 from 10 mice per group, **P < 0.001 vs. AS3d and AS3d + hM3D groups), and the total activity distance of the acute stress group did not differ from that of the control group (*P* > 0.05 vs. AS3d and AS3d + hM3D groups). (**E**) Time in light zone increased after the activation of PV-INs under acute stress conditions (n = 10 from 10 mice per group, ***P < 0.001 vs. AS3d and AS3d + hM3D groups). (**F**) Chronic stress model was established 14 days after virus injection, and behavioural monitoring of CNO activation was performed 15 min before intraperitoneal injection. (**G**) Time in center increased after the activation of PV-INs under chronic stress conditions (n = 10 from 10 mice per group, ***P < 0.001 vs. CS14d and CS14d + hM3D groups), and the total activity distance of the chronic stress group did not differ from that of the control group (*P* > 0.05 vs. CS14d and CS14d + hM3D groups). (**H**) Time in light zone increased after the activation of PV-INs under chronic stress conditions (n = 10 from 10 mice per group, ***P < 0.001 vs. CS14d and CS14d + hM3D groups). Data are presented as mean ± SEM.

To investigate the regulatory role of PV-INs on glutamatergic neurons in the BLA under acute and chronic stress, PV-INs were chemically regulated under physiological, acute stress and chronic stress conditions, and the behavioural responses of mice were assessed through the open field test and light–dark box shuttle test. Initially, physiological inhibition of PV-INs was observed. In the open field test, the percentage of active time in the central area decreased in CON + hM4D(CNO) group (Unpaired *t test*, *t* = 2.918, *P* < 0.05), with no significant difference in the total movement distance between the three groups (Fig. 5A, Unpaired *t test, t* = 1.293, *P* = 0.2250). Similarly, the light–dark box shuttle experiment demonstrated that the percentage of light box activity time of mice decreased after inhibition of PV-INs in BLA (Fig. 5B, Unpaired *t test*, *t* = 6.140, *P* < 0.0001). This suggests that the physiological inhibition of PV-INs induces anxiety-like behaviour in mice. Subsequently, we established an acute stress model to observe the behavioural changes of activated PV-INs after acute stress (Fig. 5C). The open field test revealed that the percentage of active time in the central region of activated PV-INs increased in AS3d + hM3d (CNO) group after acute stress, (Unpaired *t test*, *t* = 3.818, *P* < 0.001), with no significant difference in the total movement distance between the three groups (Fig. 5D, Unpaired *t test*, *t* = 0.2764, *P* = 0.7915). Moreover, the percentage of active time in the light box of mice increased in AS3d + hM3d (CNO) group, suggesting that the activation of PV-INs can alleviate anxiety-like behaviours induced by acute stress to a certain extent (Fig. 5E, Unpaired t test, *t* = 9.455, *P* < 0.0001). In the context of chronic stress, the percentage of activity time in the central region of activated PV-INs in CS14d + hM3D (CNO) group after chronic stress was reduced (Fig. 5G, Unpaired *t test*, *t* = 2.454, *P* < 0.05), and there was no significant difference in the total movement distance between the three groups (Fig. 5H, Unpaired *t test*, *t* = 1.164, *P* = 0.2888). Moreover, the percentage of active time in the light box of mice decreased in CS14d + hM3D (CNO) group (Fig. 5H, Unpaired *t test*, *t* = 7.329, *P* < 0.0001). These findings demonstrate that the inhibition of PV-INs induces anxiety-like behaviour, while activation of PV-INs can alleviate anxiety-like behaviours induced by acute stress but exacerbate them under chronic stress conditions.

## Discussion

Stress is a systemic non-specific adaptive response that occurs when the body is stimulated by internal and external environmental factors as well as social and psychological factors. There are mature models for the study of stress. At present, the main models of stress are bondage, forced swimming, foot electric shock, water fasting and unpredictability^24,25^. According to the different responses of the body, stress is usually divided into three categories: physical stress, psychological stress and compound stress^26,27^. Ice water forced swimming has both physical and psychological stress. In this study, the acute stress model of ice-water forced swimming was used to simulate the extreme compound stress in judicial practice. The classical model of unpredictable stress was adopted for chronic stress. We used common open field test and light–dark box shuttle test to evaluate the anxiety-like behavior of mice, and the results showed that animal model was successfully constructed.

The proper number of inhibitory neurons is the material basis for maintaining the function of amygdala^18,28,29^. In the study, immunohistochemical analysis revealed a significant sensitivity of PV-INs to stress damage, with a decrease observed in their numbers in the BLA following both acute and chronic stress exposure. Similar downregulation of PV-IN expression has been reported in the postmortem cerebral cortex of various autistic individuals^29^ and also in disorders like dementia and various psychiatric disorders^30–32^. Studies have shown that PV-INs are prone to damage^33–37^, which could be related to the high metabolic demand of PV-INs under sustained and severe stress. Furthermore, the metabolic requirements of PV-INs may be depleted, causing oxidative stress that triggers pro-apoptotic signalling and cell death. It can be seen that the reduction of the number of PV-INs caused by damage is one of the main manifestations that lead to the change of mental behaviour. Stress mainly leads to stress-induced anxious behaviour by reducing the number of PV-INs and triggering disinhibition.

The hyperactivation of glutamatergic neurons in the BLA resulting from disinhibition is widely implicated in stress-induced psychiatric disorders^4,28^. Our study corroborates this notion, demonstrating that both acute and chronic stress leads to a decrease in PV-INs in the BLA, accompanied by decreased inhibition of glutamatergic neurons as detected by electrophysiological techniques. This reduced inhibition manifests as increased disinhibitory discharge of glutamatergic neurons and heightened excitability, ultimately contributing to anxiety-like behaviour changes in mice. Furthermore, chemical regulation of PV-IN activity revealed that the inhibition of PV-INs in the physiological state enhances glutamatergic neurons excitability and induces anxiety-like behaviour. Conversely, activation of PV-INs after acute and chronic stress reduces glutamatergic neurons excitability, alleviating stress-induced anxiety-like behaviour. Additionally, previous studies have shown that PV-INs play a role in fear learning, wherein excitatory PV-INs enhance fear learning and silenced PV-INs impair fear learning^38^. These studies underline the significance of PV-INs in regulating mental and behavioural responses by affecting fear learning. Furthermore, the chemogenetic activation of PV-INs inhibits the firing rate of peripheral glutamatergic neurons, which in turn produces antiepileptic effects through ion-gated channels, GABAa receptors^20,39–41^. PV-INs possess certain properties that enable them to efficiently regulate glutamatergic neurons. Moreover, PV-INs fire and repolarise more rapidly, which causes them to exhibit fast-spiking action potential firing patterns. Additionally, they also have a low threshold for producing spikes and can fire rapidly without relative fatigue^42,43^. Therefore, studies have shown that acute and chronic stress reduces the number of PV-INs, resulting in the weakened inhibition of PV-INs, which is manifested as excessive activation of disinhibitory excitation of glutamatergic neurons, leading to anxiety-like behaviour.

The current lack of precise treatments for anxiety disorders presents a significant challenge^3,44^. Benzodiazepines are commonly used as the first-line treatment for anxiety behaviour as they promote the binding of GABA to GABAa receptors, resulting in an increase in the opening frequency of chloride channels and leading to hyperpolarisation, which consequently decreases the excitability of glutamatergic neurons. However, there are many sub-classes of GABAergic interneurons with complex mutual regulation, resulting in poor targeting and unsatisfactory efficacy of benzodiazepines. Moreover, the tolerability of benzodiazepines and the risk of addiction also limit their long-term use^45^. In this study, the transfection of PV-INs in the BLA with hM3Dq and activation of PV-INs with CNO were effective in alleviating anxiety behaviour caused by acute and chronic stress, no side effects were found. Similarly, in a study of epilepsy, hM3Dq was used to transfer PV-INs into the hippocampal epileptic foci, and CNO activation increased the firing rate of parvalbumin neurons and inhibited the firing rate of glutamatergic neurons, thereby alleviating epileptic seizures. Overall, this study provides an experimental basis for the development of new pharmacogenetic treatments for stress-induced anxiety behaviour.

While this study did not comprehensively explore the interactions between different inhibitory interneurons and excitatory glutamatergic neurons within neural microcircuits, it contributes to our understanding of the role of PV-INs in acute and chronic stress-induced anxiety behaviour. By elucidating these mechanisms, our findings offer insights into potential therapeutic targets for anxiety behaviour and pave the way for further research in this area.

## Methods and materials

### Animals

One hundred and sixty-five male C57BL/6N mice, aged 6–7 weeks and weighing (20 ± 2) g, were procured from Beijing Vital River Laboratory Animal Technology Co., Ltd. Mice were housed in a controlled environment with constant temperature and humidity, maintained on a 12 h light/dark cycle and provided ad libitum access to food and water. These mice were adapted to the environment for at least 7 days prior to experiment. The mice were handled for 5 min every day before the experiment to minimize fear of the environment and the experimenter. Mice were housed in groups of five in cages and categorised into three groups: control group (CON group), acute stress group (AS3d group) and chronic stress group (CS14d group). This experimental protocol was approved by the Laboratory Animal Management Committee of Hebei Medical University (approval number: 20223011). All experimental procedures adhered to the Animal Research: Reporting of In Vivo Experiments (ARRIVE) guidelines (https://arriveguidelines.org).

### The establishment of a stress model

Two stress models were employed: acute stress and chronic stress. We chose highly intense stressors to simulate judicial practice. Acute stress was induced using a combination of classical restraint and ice-water swimming^18,46^. Mice were restrained in a 2.1 × 1.2 × 2.3 cm tube for 6 h daily and subsequently subjected to a 3-min swim in ice water for three days. Chronic stress utilised the classical chronic unpredictable mild stress model^19^, incorporating seven different stress modes. These stressors included the following: mice were confined to a 2.1 × 1.2 × 2.3 cm restraint tube for 6 h on the first day; mice were exposed to a humid environment with wet bedding material for 24 h on the second day. On the third day, the vascular clamp was applied to the distal 1/3 of the tail of mice for 1 min. Then, the mice were placed on a shaker for 1 h and the rotational speed was maintained at 130 ± 5 r/h on the fourth day, and were immersed in ice water to swim for 3 min on the fifth day. On the last two days, the mice were exposed to the reversed light–dark cycle and fasting/water prohibition for 24 h respectively. The stress pattern of the last seven days was the same as that of the first seven days, in random order. The chronic stress lasted for 14 days.

### Light and dark box shuttle test and open field test

To assess anxiety-like behaviour in mice, an open field test was conducted on the first day following acute stress for 3 d and chronic stress for 14 d. The experiments were performed in the photoperiod of the mice. Prior to the experiment’s commencement, the mice were habituated for more than 30 min in the monitoring room. The mice were placed in a test chamber of 40 × 40 × 30 cm size, back to the observer, and their movements were recorded every 5 min. After the experiment concluded, the test chamber was disinfected and cleaned with alcohol, and the mice were put back into their original cage. The ability to explore the central region was decreased when the anxiety-like behaviour occurred in mice. The indexes were selected as percentage of central region active time (%) and total active distance (cm).

The light–dark box shuttle experiment was conducted on the second day following acute stress for three days and chronic stress for 14 days. One side of the light–dark box is equipped with light source of 400 lx, the size is 20 × 30 × 25 cm, called the bright box, and the other side is the black box, the size is 20 × 20 × 25 cm, with a small door (7.5 × 7.5 cm) between the two areas to allow the mice to walk freely. Two infrared cameras accurately recorded the residence time and route of the mice in each region. Prior to the experiment’s commencement, the mice were habituated to the monitoring room for more than 30 min. The mice were placed in the bright box for 5 min with their back to the observer. Notably, when the anxiety-like behaviour occurred in mice, the exploration ability of the light box was decreased, and the index was selected as the percentage (%) of the light box activity time.

### Immunohistochemical analysis

After the mice were sacrificed, brain tissue was collected, fixed, dehydrated and embedded in wax blocks for paraffin sectioning. Sections were oven-dried at 62℃ for 40 min, followed by dewaxing, hydration, antigen repair, endogenous peroxidase and serum blocking. Primary antibody Anti-parvalbumin (1:100, ab181086, Abcam) was added drop by drop to the samples, which were then incubated at 4℃ overnight. The next day, following washing with 1 × PBS, a reaction enhancement solution was added to enhance the enzyme-labelled goat anti-mouse/rabbit IgG polymer, followed by DAB colour development, hematoxylin nucleation, hydrochloric acid alcohol differentiation, dehydration, xylene soaking and neutral gum sealing. The number of PV^+^ cells were counted in bilateral BLA at 200× magnification. Five mice from each group were used to morphological observation and data analysis^18^. The largest amygdala area was accurately identified according to the stereotaxic atlas. Serial section technique was used to select one of every five sections for a total of three sections per mice. The mean number of positive cells in each mouse was calculated by two independent observers who were blinded to the experimental conditions.

### Immunofluorescence

Paraffin sections underwent 40 min of oven drying at 62℃, followed by dewaxing, hydration, antigen repair, goat serum sealing, Anti-parvalbumin (1:100, ab181086, Abcam) drip and overnight incubation at 4℃. On the following day, sections were washed with 1× PBS and incubated with fluorescent Anti-rabbit IgG (1:500, Alexa FluorTM 488 donkey) at 37℃ to label PV-INs. Confocal laser microscopy was utilised after DAPI staining to observe positive PV-IN expression. Additionally, the co-expression of glutamatergic neurons and c-fos in the BLA was examined through fluorescent double labelling. Primary antibodies included Anti-CAMKII (1:200, ab134041, Abcam) and Anti-c-fos (1:200, ab208942, Abcam), with secondary antibodies including Anti-rabbit IgG (1:500, Alexa FluorTM 488 donkey) for glutaminergic neurons and Anti-mouse IgG (1:500, Alexa FluorTM 568 donkey) for activated neurons. The numbers of PV^+^, CAMKII^+^, c-fos^+^, and CAMKII^+^-c-fos^+^ cells were counted in bilateral BLA at 200× magnification. The mean number of positive cells in each mices was calculated by two independent observers who were blinded to the experimental conditions.

### Stereotaxic injection of virus into the brain

Mice were fully anaesthetised with 2% sodium pentobarbital and secured in a brain stereotaxic device. Following scalp shaving and midline incision, coordinates for the anterior and posterior fontanel were set. Based on this coordinate position, the coordinates of bilateral BLA were set (anteroposterior, 1.46 mm; mediolateral, ± 2.86 mm; dorsoventral: 4.79 mm). After drilling, adeno-associated viruses AAV-VGLUT2-EGFP (AAV2/9, 2.00 × 10^12^vg/ml, 100 nl, BrainVT A (Wuhan) Co., Ltd), AAV-PV-INs-CRE (AAV2/9, 2.00 × 10^12^vg/ml, 100 nl, BrainVT A (Wuhan) Co., Ltd) and AAV-hsyn-DIO-hM4Di-mCherry (AAV2/9, 2.00 × 10^12^vg/ml, 100 nl, BrainVT A (Wuhan) Co., Ltd)/AAV-hsyn-DIO-hM3Dq-mCherry (AAV2/9, 2.00 × 10^12^vg/ml, 100 nl, BrainVT A (Wuhan) Co., Ltd) were injected into the BLA on both sides through a calibrated glass microelectrode connected to a syringe (1 ml, Hamilton Company, USA) using an infusion pump (RWD Life Science, Shenzhen, China) at the rate 20 nl/min . After injection, the syringe was retracted, the scalp was sutured and the area was disinfected. Mice were returned to their original cages for two weeks of normal feeding before subsequent follow-up experiments, including CNO injection for behavioural and patch-clamp experiments.

### Electrophysiology

#### Section preparation

Mice were fully anaesthetised with 2% sodium pentobarbital and fixed. Their brains were harvested on ice after perfusion with an artificial cerebrospinal fluid (aCSF) cutting solution (92 mM N-methyl-d-glucamine diatrizoate, 1.2 mM NaH2PO4, 30 mM NaHCO3, 20 mM HEPES, 25 mM glucose, 5 mM ascorbate, 2 mM thiourea, 3 mM Gluuvate, 2.5 mM KCL, 10 mM MgSO4 and 0.5 mM CaCL2). Brain tissue was placed in the aCSF solution, which was precooled and oxygenated for more than 1 h, for 1-2 min. Slices were made along the coronal plane on a Leica VT1200S vibratome (Leica, Germany) with a thickness of 300 μm, a slice speed of 0.2 mm/s and an amplitude of 1.45 mm. Brain slices were incubated in holding aCSF solution, comprising HEPES and oxygenated for 1 h. The holding aCSF solution comprised 92 mM NaCl, 1.2 mM NaH_2_PO_4_,30 mM NaHCO_3_, 20 mM HEPES, 25 mM glucose, 5 mM ascorbate, 2 mM thiourea, 3 mM Gluuvate, 2.5 mM KCl, 1 mM MgCl_2_ and 2 mM CaCl_2_.In the chemogenetic modulation experiment, CNO needs to be injected intraperitoneally 15 min in advance for subsequent steps such as anaesthesia.

#### Electrophysiological recordings of glutamatergic neurons in the BLA

The recording aCSF solution comprised 126 mM NaCl, 1.2 mM NaH_2_PO_4_, 26 mM NaHCO_3_, 11 mM glucose, 3 mM KCl, 1.5 mM MgCl_2_ and 2.4 mM CaCl_2_. The frontal fluorescence phase contrast microscope revealed BLA at low power (5 ×). The BLA was placed in the centre of the visual field and the high-power lens (40 ×) for utilised for observation. After adjusting the focal length, fluorescence was used to identify the fluorescent-labelled vertebral neurons, recording full rather than swollen neurons with clear boundaries. Whole cell patch recording was performed using borosilicate glass pipettes drawn by a P-100 micropipette puller (Sutter Instruments, USA). Electrical signals were recorded using Clampex 10.1 and Axon 700B chip amplifiers (Axon Instruments, USA). To measure the evoked action potential, cells were held at near resting potential (− 60mv) under a current clamp. Sequential current injections were applied every 400 ms and ranged from 0 to 400 pA with 20 pA increments each time for a total of 11 sweeps. An action potential (AP) was evoked after injecting a suprathreshold stimulating current. Each cell was recorded at least thrice, with 3–5 cells being recorded per brain slice, and each set of data was from 5–6 mice. Clampfit 10.7 was used to analyse the AP data and record the frequency or number of cell APs.

### Statistical analysis

All experimental data are presented as mean ± SEM. Data analysis was performed using GraphPad Prism 8.1 software. Statistical differences between two groups were assessed using Student’s t-test, while comparisons among three or more groups were evaluated using ANOVA followed by Bonferroni multiple comparisons. GraphPad Prism 8.1 software was utilised to generate statistical and electrophysiological figures. A p-value of less than 0.05 indicated statistical significance.

## Conclusions

Collectively, acute and chronic stress adversely affect PV-INs in the BLA, diminishing their numbers and resulting in glutamatergic neurons disinhibition, thereby enhancing glutamatergic neurons excitability and precipitating anxiety behaviour. These findings underscore the pivotal role of PV-INs in regulating glutamatergic neurons activity in stress-induced anxious behaviour. This study provides a novel target for stress anxiety disorder treatment and also provides a theoretical basis for compensation for anxiety disorders caused by severe stress.

## Data Availability

The datasets recorded and analyzed during the current study are available from the corresponding author on reasonable request.
